# An exploratory analysis of disulfidptosis-related gene signatures in minimal change disease identifies metabolic and immune associations

**DOI:** 10.3389/fcell.2026.1790068

**Published:** 2026-06-19

**Authors:** Jiahui Li, Quhuan Li, Yue Shen, Chaohong Nie, Jiao Li, Fengxia Zhang

**Affiliations:** 1 First Affiliated Hospital of Gannan Medical University, Ganzhou, China; 2 Guangdong Engineering Research Center of Low-Carbon Synthetic Biotechnology, School of Biology and Biological Engineering, South China University of Technology, Guangzhou, China; 3 The First Clinical Medical College of Gannan Medical University, Ganzhou, China

**Keywords:** minimal change disease, disulfidptosis, machine learning, molecular groups, bioinformatics

## Abstract

**Aim:**

We aimed to investigate the association between genes related to disulfidptosis—a form of cell death caused by aberrant disulfide stress and cytoskeletal collapse—and the molecular features of minimal change disease (MCD), the leading cause of primary nephrotic syndrome.

**Methods:**

Data from the GeneCards and Gene Expression Omnibus (GEO) databases were integrated to systematically analyze disulfidptosis-related genes in MCD. Machine learning approaches—including generalized linear model (GLM), support vector machine (SVM), random forest (RF), and extreme gradient boosting (XGBoost)—pinpointed four hub genes. These genes were used to construct a diagnostic nomogram. Molecular groups were defined by consensus clustering, and pathway alterations were explored through gene set variation analysis and gene set enrichment analyses. Gene expression was validated by immunohistochemistry (IHC) and immunofluorescence (IF).

**Results:**

The diagnostic model based on *FLNC*, *GYS1*, *INF2*, and *MYH11* demonstrated exploratory discriminatory performance (AUC = 0.839). Consensus clustering based on the four hub genes suggested two potential molecular groups (C1 and C2), and their differences in pathway enrichment and immune characteristics were explored. *MYH11* expression was markedly higher in the C2 group and correlated with cytoskeletal remodeling and immune modulation. Experimental validation confirmed significant upregulation of *MYH11* in MCD.

**Conclusion:**

This study indicates a potential link between disulfidptosis-related genes and MCD, and presents an exploratory diagnostic and molecular classification system that requires further validation in larger cohorts.

## 1 Introduction

Minimal change disease (MCD) is a leading cause of nephrotic syndrome, particularly in children, accounting for 70%–90% pediatric cases and 10%–15% of adult cases (Yan et al., 2020; Cameron, 1996). Although MCD typically exhibits high glucocorticoid sensitivity, with remission rates exceeding 80% during initial episodes (Kidney Disease: Improving Global Outcomes KDIGO Glomerular Diseases Work Group, 2021), its pathogenesis remains poorly understood. Adult patients, in particular, experience high relapse rates (>50%) (Shinzawa et al., 2014), emphasizing the urgent need for a deeper mechanistic understanding and more effective therapeutic strategies.

MCD is recognized as a podocytopathy primarily driven by podocyte injury, leading to significant proteinuria and impaired glomerular filtration rate. Characteristic pathological features include podocyte foot process effacement and disruption of the glomerular filtration barrier, which are frequently associated with cytoskeletal dysregulation and loss of nephrin function (Watts et al., 2022). Antinephrin autoantibodies have been detected in approximately 29% of patients (Shen and Fillatreau, 2015). Recent studies have revealed that disulfidptosis contributes to cell death under glucose-deprived conditions, where SLC7A11-mediated excessive cystine uptake, coupled with NADPH deficiency, triggers disulfide stress, aberrant actin crosslinking, and irreversible cytotoxic damage (Badgley et al., 2020; Zheng et al., 2023; Liu et al., 2020). This cell death pathway is distinct from ferroptosis and highlights the pivotal role of metabolic and redox homeostasis in maintaining podocyte integrity, providing novel insights into MCD pathogenesis.

The integrity of the glomerular filtration barrier depends critically on podocyte function, where dynamic nephrin phosphorylation and cytoskeletal stability are tightly regulated by redox homeostasis (Watts et al., 2022; Pozzi, 2012). Disruption of this balance can trigger disulfide stress through interconnected mechanisms, including autoantibody-mediated nephrin dysfunction, hyperactivation of the FAK/JAK–STAT signaling pathway, endothelial glycocalyx damage that compromises the barrier function, and immune dysregulation marked by B-cell-driven T cell resistance (Uchida et al., 2008; Sein et al., 2000; Jamin et al., 2018). Together, these processes contribute to the breakdown of glomerular filtration function in MCD. Although the molecular mechanisms of MCD have been partially elucidated and the cascade of disulfidptosis has been characterized, the potential link between disulfidptosis and MCD remains entirely unexplored. In this study, we investigated the potential link between disulfidptosis-related genes and the molecular mechanisms underlying MCD, aiming to deepen the understanding of its pathogenesis.

**FIGURE 1:**
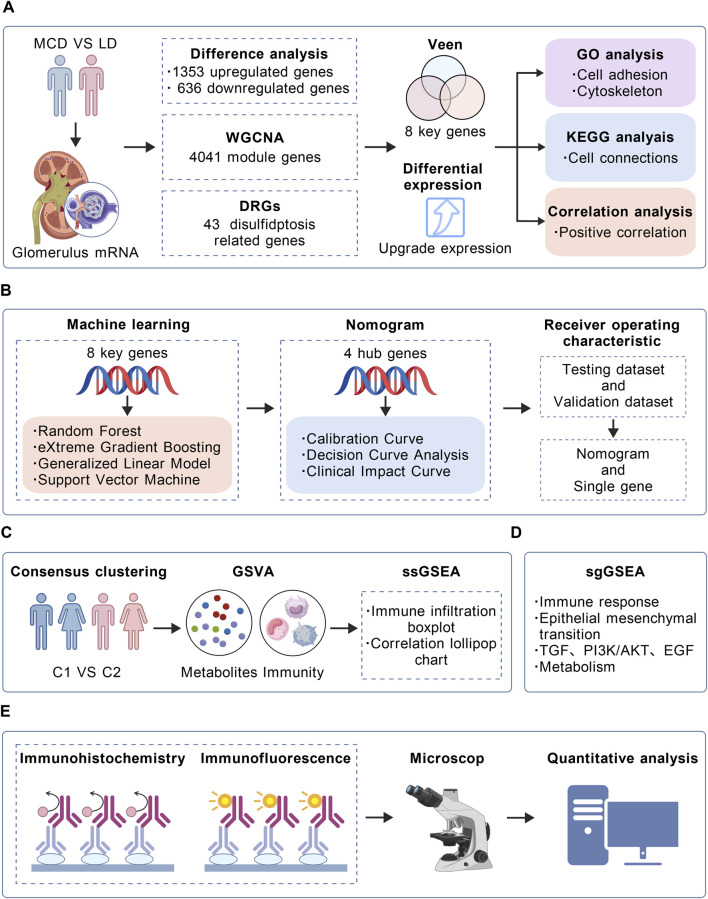
Technical workflow of the study. **(A)** Screening workflow for key genes linking minimal change disease (MCD) and disulfidptosis. **(B)** Construction of a diagnostic prediction model. **(C)** Group identification analysis. **(D)** Functional analysis of hub genes. **(E)** Experimental validation.

Figure 1 presents the experimental design and methodological framework of this investigation, which was generated using BioGDP (Jiang et al., 2025).

## 2 Materials and methods

### 2.1 Data collection and collation

Three microarray datasets (GSE108109, GSE200828, and GSE104948) were obtained from the Gene Expression Omnibus (GEO) database (Edgar et al., 2002). After excluding non-glomerular samples and those from patients with other chronic kidney diseases, the training cohort comprised 40 MCD samples and 30 normal controls (GSE108109:16 MCD, 6 normal; GSE200828:19 MCD, 6 normal; GSE104948-GPL22945:5 MCD, 18 normal). An independent validation cohort included nine MCD and three normal samples from the GPL24120 platform of GSE104948. Probe annotation was conducted using the org.Hs.e.g.db R package. For genes with multiple corresponding probes, mean expression values were calculated. Only genes consistently detected across all datasets were retained for further analyses. Batch effects were corrected using the Limma R package (Ritchie et al., 2015). A total of 43 disulfidptosis-related genes were identified through a comprehensive literature review and a GeneCards database search (https://www.genecards.org/) include CYFIP1,EMILIN1,AJAP1,TM9SF2,FLNA, FLNB,MYH9,TLN1,ACTB,MYL6,MYH10,CAPZB, DSTN,IQGAP1,ACTN4,PDLIM1,CD2AP,INF2,SLC7A11,GLUT, NCKAP1,SLC3A2,GYS1,NDUFA11,RAC1,RPN1,NDUFS1,NUBPL, LRPPRC,OXSM, NADPH,BRK1,WASF2,ABI2,PRDX1,ACSL4,BAK1,TLN2,FLNC,MYL6B,DBN1,ACTN1,MYH11.

### 2.2 Weighted correlation network analysis (WGCNA)

WGCNA was conducted to identify gene modules pivotal to the pathogenesis and progression of MCD (Zhang and Horvath, 2005; Langfelder and Horvath, 2008). Pearson correlation coefficients were calculated to construct the correlation matrix. To ensure network robustness, a scale-free topology fitting index (*R* ^2^) > 0.85 was applied. Using the pickSoftThreshold function in the WGCNA R package, the optimal soft-thresholding power (β) was selected to quantify network connectivity and establish a scale-free co-expression network. This network was subsequently transformed into a topological overlap matrix (TOM), and the corresponding dissimilarity measure (1-TOM) was computed. Using TOM-based dissimilarity, average-linkage hierarchical clustering identified gene modules, each containing at least 50 genes. After determining an appropriate cutting height and merging highly similar modules, key modules were identified. Finally, gene–module correlations were calculated and visualized using scatter plots to identify core modules most closely associated with the traits of interest.

### 2.3 Identification of differentially expressed genes (DEGs)

We used the Limma R package to identify DEGs that set MCD samples apart from normal ones.16 To qualify as truly significant, genes needed to exhibit both with |logFC| > 0.3 and p-value <0.05. A volcano plot served as our visual aid to map out these distinctive genetic markers.

### 2.4 Enrichment analysis

Gene ontology (GO) enrichment analysis was performed using the *enrich*GO function of the clusterProfiler R package in conjunction with the org.Hs.eg.db package to elucidate the functional roles of key genes (Yu et al., 2012). The analysis covered three major categories: biological processes (BP), cellular components (CC), and molecular functions (MF). In addition, Kyoto Encyclopedia of Genes and Genomes (KEGG) pathway enrichment analysis was conducted using the *enrichKEGG* function of the clusterProfiler and org.Hs.e.gdb packages (Yu et al., 2012), with a significance threshold of p < 0.05. The results were visualized using the *ggplot2* and *GOplot* R packages.

### 2.5 Machine learning algorithm

To identify hub genes significantly associated with MCD progression, four machine learning models were used. Data partitioning was performed using the caret R package. The data splitting strategy involved randomly assigning samples to the training and validation groups in a ratio of 7:3. The createDataPartition function was utilized to conduct random sampling, ensuring that the samples were representative of the overall distribution in both groups. Four machine learning models [generalized linear model (GLM), support vector machine(SVM), random forest(RF), and extreme gradient boosting (XGBoost)] were then trained and evaluated using repeated 10-fold cross-validation to reduce the risk of overfitting (Qiao et al., 2024). Each model’s efficacy was assessed through receiver operating characteristic (ROC) curve analysis and residual distribution assessment (Hanley and McNeil, 1982). Hub genes were then selected based on the most effective model.

### 2.6 Construction of the nomogram model

To predict the risk of MCD, a nomogram was constructed using four hub genes identified via machine learning (Balachandran et al., 2015). The model was developed using the root mean square (RMS) R package, based on a multivariate regression framework (Zhang and Kattan, 2017). Each gene was assigned a score proportional to its contribution to MCD risk, and the total score was used to estimate the probability of disease occurrence. Model performance was evaluated using calibration curves generated with the rms and ggplot R packages to verify correspondence between expected and actual risks (Sadatsafavi et al., 2022). To further assess model calibration and overfitting, we performed bootstrap-based calibration curve analysis with 500 resamples. The ggDCA R package assessed net clinical benefit across multiple threshold probability points through decision curve analysis (DCA) (Van Calster et al., 2018), and a clinical impact curve (CIC) was generated with the rmda R package to illustrate the concordance between predicted high-risk cases and actual outcomes (Janse et al., 2024).

### 2.7 ROC analysis

The nomogramFormula R package was used to calculate nomogram scores for each patient, and the pROC R package was used to generate ROC curves based on the nomogram model to evaluate its discriminatory performance (Robin et al., 2011; Obuchowski and Bullen, 2018). A higher area under the curve (AUC), ranging from 0.5 to 1, indicated greater model accuracy. Nomogram forecasting accuracy was further assessed by comparing its ROC curves with those derived from single-gene analyses. Samples annotated with GPL24120 in the GSE104948 dataset were used as an external validation cohort.

### 2.8 Consensus cluster analysis

Consensus clustering was performed using the ConsensusClusterPlus R package to classify patients with MCD into distinct molecular groups (Wilkerson and Hayes, 2010). Principal component analysis (PCA) was then applied to validate the separation of these groups (Ben Salem and Ben Abdelaziz, 2021). Differential expression boxplots were generated using the ggpubr R package to compare hub gene expression levels across the identified groups.

### 2.9 Gene set variation analysis (GSVA)

GSVA was performed to assess differences in pathway activity across MCD groups using the gene expression matrix (Hänze et al., 2013). GO pathway gene sets from the C5 collection of the MSigDB database (https://www.gsea-msigdb.org/gsea/msigdb) were used as reference sets. The GSVA algorithm transformed gene expression data into pathway enrichment scores. A contrast matrix was constructed between the two groups, and the Limma R package was used to identify significantly enriched pathways with p < 0.05(16). Heatmaps and volcano plots were generated in R, and bar charts illustrating the top 20 differentially expressed pathways were created using the ggplot2 R package.

### 2.10 Immune infiltration analysis

A comprehensive analysis of the immune microenvironment was conducted to characterize the immune features of patients with MCD. Single-sample gene set enrichment analysis (ssGSEA) was performed using the GSVA R package to quantify infiltration levels of 28 immune cell types in MCD samples (Hänze et al., 2013; Wang et al., 2022). Boxplots were generated to visualize differences in immune cell infiltration across groups. Spearman’s correlation analysis examined associations between the four identified core genes and immune cells, with the results presented in Laplace plots.

### 2.11 Single-gene gene set enrichment analysis (sgGSEA)

sgGSEA was performed to investigate the regulatory pathways and biological functions associated with the four identified hub genes (Zhou et al., 2023). Statistical significance was defined as p < 0.05.

### 2.12 Immunohistochemistry (IHC)

Renal tissues were fixed in 4% paraformaldehyde (PFA) and embedded in paraffin. Sections were deparaffinized in xylene, rehydrated through a graded ethanol series, and rinsed with phosphate-buffered saline (PBS). Endogenous peroxidase activity was quenched by treatment with 3% H_2_O_2_ at 37 °C. Following antigen retrieval, non-specific binding sites were blocked using 3% bovine serum albumin (BSA). Sections were incubated overnight at 4 °C with an anti-MYH11 antibody (Proteintech Group Inc., Chicago, IL, United States; 60222-1-Ig, 1:500) to assess *MYH11* expression in renal tissue. After incubation with a species-matched secondary antibody, IHC detection was performed using DAB, followed by counterstaining with hematoxylin. Sections were then dehydrated, cleared, and mounted. Images were acquired using a microscope and semi-quantitatively analyzed using ImageJ (version 1.54p) (Taylor, 2000).

### 2.13 Immunofluorescence (IF)

Tissue sections were fixed in 4% PFA for 15 min, blocked with 3% BSA, and incubated overnight at 4 °C with primary antibodies against MYH11 (Proteintech Group Inc, Chicago, IL, United States; 60222-1-Ig, 1:100) and nephrin (Proteintech Group Inc, Chicago, IL, United States; 22912-1-AP, 1:1000). Following the washing step, the sections underwent a 1-h incubation at 37 °C with species-specific secondary antibodies, after which they were stained with 4′,6-diamidino-2-phenylindole (DAPI). Fluorescence microscopy was used to capture the images, and ImageJ software was used to quantify the fluorescence intensity (Odell and Cook, 2013; Im et al., 2019; Tan et al., 2020).

### 2.14 Statistical analysis

All statistical analyses were performed using R software (version 4.3.3) and GraphPad Prism (version 9.5.0). Data are presented as mean ± SEM. Statistical significance was defined as p < 0.05, with significance levels indicated as follows: *p < 0.05 (*), **p < 0.01 (**), ***p < 0.001 (***), and ****p < 0.0001 (****).

## 3 Results

### 3.1 Screening of disulfidptosis-related genes and identification of MCD co-expression gene modules

**FIGURE 2:**
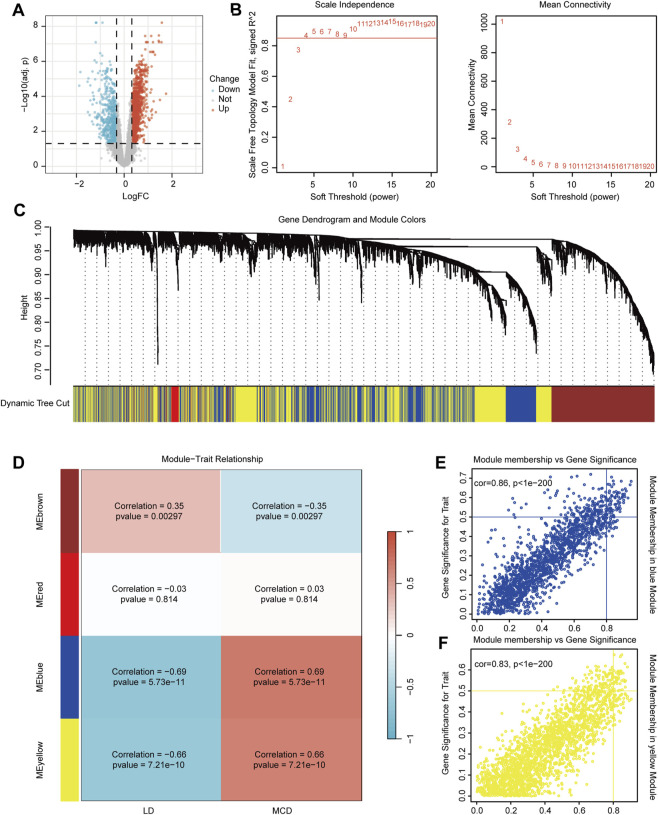
Screening of differentially expressed genes (DEGs) and identification of co-expression modules in minimal change disease (MCD). **(A)** Volcano plot of DEGs between the MCD and normal samples. **(B)** Network topology analysis for various soft thresholding powers. **(C)** Gene dendrogram derived from average linkage hierarchical clustering. **(D)** Heatmap of module-trait correlations with MCD. **(E)** Scatter plot of gene significance *versus* module membership in the blue module. **(F)** Scatter plot of gene significance *versus* module membership in the yellow module.

To ensure the reliability of the integrated dataset, batch-effect correction was performed using the Limma package. As shown in Supplementary Figure S1, batch effects were effectively removed while the biological differences between MCD and normal samples were preserved. Following this correction, comparison between patients with MCD and normal samples revealed a total of 1989 DEGs, including 1353 upregulated and 636 downregulated genes (|logFC| > 0.3, p-value <0.05). A volcano plot was used to visualize genes with significant expression differences between MCD and normal groups (Figure 2A). WGCNA was conducted using the integrated gene expression matrix to identify key modules associated with MCD. Using a scale-free fitting index *R* ^2^ > 0.85 and high average connectivity, the optimal soft threshold (β) was set at 4 to construct a biologically meaningful scale-free network (Figure 2B). Hierarchical clustering analysis and dynamic tree-cutting identified four key modules (Figure 2C). Connections between modules and clinical features was also examined, and the module–trait correlation analysis revealed that the blue module had a correlation coefficient of 0.69 with the disease group, whereas the yellow module had a coefficient of 0.66 (p-value <0.05), both showing significant positive correlations with the disease group. These two modules were selected for subsequent analyses (Figure 2D).

Scatter plots showed notable associations between the genes in these two modules and MCD, with correlation coefficients of 0.86 and 0.83, respectively (Figures 2E,F). Combining the genes from both modules resulted in a set of 4,041 key module genes.

### 3.2 Identification of key genes and functional enrichment analysis

**FIGURE 3:**
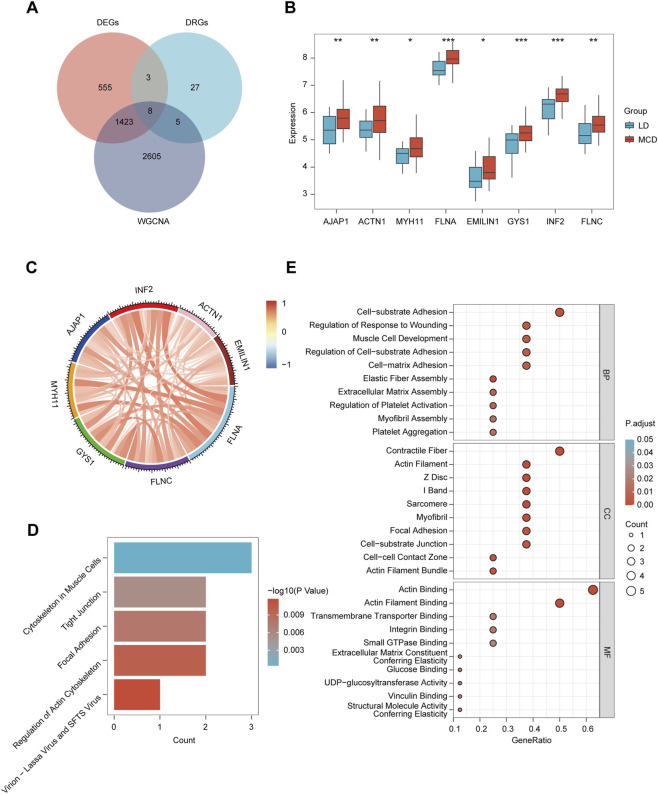
Identification of key disulfidptosis-related genes and functional enrichment analysis in minimal change disease (MCD). **(A)** Venn diagram showing intersections among differentially expressed genes (DEGs), Weighted correlation network analysis (WGCNA) module genes, and 43 disulfide-related genes. **(B)** Box plots displaying the differential expression patterns of eight key genes between MCD and normal samples. **(C)** Circos plot illustrating correlations among the eight key genes. **(D)** Bubble plot of the GO enrichment analysis for the eight key genes. **(E)** Bar plot of the Kyoto Encyclopedia of Genes and Genomes (KEGG) pathway analysis for the eight key genes.

The intersection between the previously identified module genes and disulfidptosis-related genes was determined using a Venn diagram, which revealed eight overlapping genes with potential as candidate transcriptomic markers for MCD. These genes—*AJAP1*, *ACTN1*, *MYH11*, *FLNA*, *EMILIN1*, *GYS1*, *INF2*, and *FLNC*—were designated as the key genes in this study (Figure 3A). Boxplots revealed significant upregulation of all eight genes in the MCD group compared with the normal group (Figure 3B). The correlation analysis revealed positive correlations among these genes, consistent with their differential expression patterns (Figure 3C). GO and KEGG enrichment analyses were conducted to clarify the biological functions of these key genes. The GO analysis identified significant enrichment in pathways associated with cytoskeleton organization, including myofibril assembly, actin–binding processes, cell–cell adhesion, and cell–matrix adhesion (Figure 3D). Similarly, KEGG analysis revealed significant enrichment in pathways related to cytoskeleton regulation and cell junctions, such as tight junction and focal adhesion pathways (Figure 3E). Additionally, to explore the broader biological context of MCD-associated pathways, we performed GO enrichment analysis on the 1,488 genes that overlapped between the WGCNA module genes and the DEGs. These analyses also revealed significant enrichment in pathways such as cell-matrix adhesion (Supplementary Figure S2). The concordance between the GO and KEGG enrichment results and the known characteristics of disulfidptosis further supports the reliability of these findings.

### 3.3 Machine learning identification of hub genes

**FIGURE 4:**
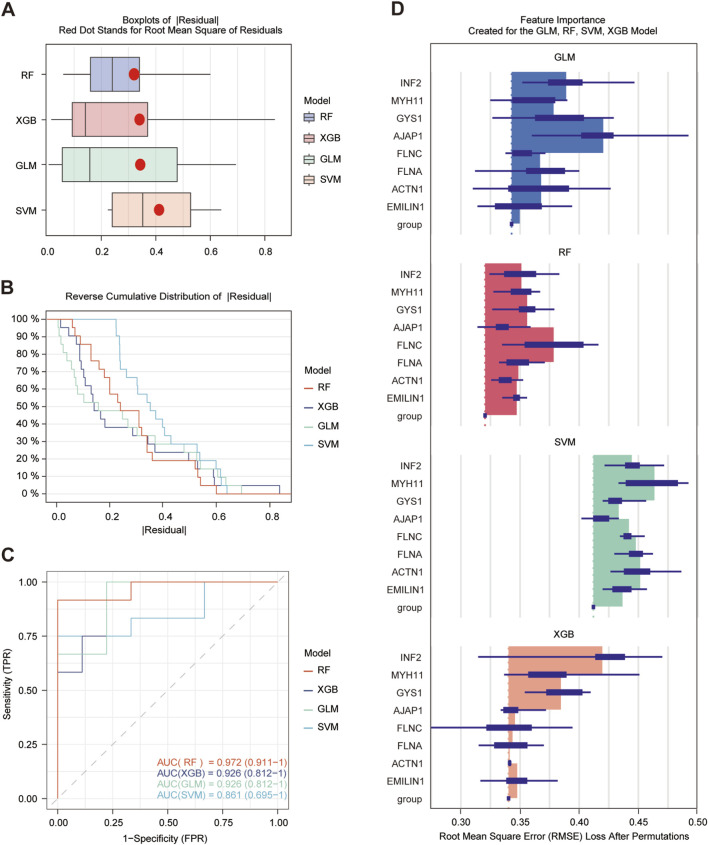
Machine learning-based screening of candidate transcriptomic markers in minimal change disease (MCD). **(A)** Residual boxplots of random forest (RF), and extreme gradient boosting (XGB), generalized linear model (GLM), support vector machine (SVM) models. **(B)** Reverse cumulative distribution of residuals for four models. **(C)** Receiver operating characteristic (ROC) curves evaluating the predictive accuracy of each model. **(D)** Bar plots of feature importance scores from the four machine learning models.

To further identify candidate transcriptomic markers associated with MCD and disulfidptosis, four distinct machine learning algorithms—XGBoost (XGB), GLM, SVM, and RF—were applied. Among these, the RF algorithm showed optimal residual distribution characteristics. Boxplots of residual distribution showed that the RF model had the lowest RMS error and a narrower interquartile range than the other three algorithms (Figure 4A). The reverse cumulative distribution plot confirmed that the RF algorithm maintained the lowest cumulative residuals in the high-error range (Figure 4B). Moreover, based on the ROC curve, the RF algorithm achieved the highest AUC = 0.972, indicating relatively better predictive performance compared with the other models (Figure 4C). Consequently, the RF algorithm was selected as the optimal model. Based on feature importance ranking, the top four genes—*FLNC*, *GYS1*, *INF2*, and *MYH11*—were identified and designated as hub genes associated with MCD and disulfidptosis (Figure 4D).

### 3.4 Construction and validation of the nomogram model

**FIGURE 5:**
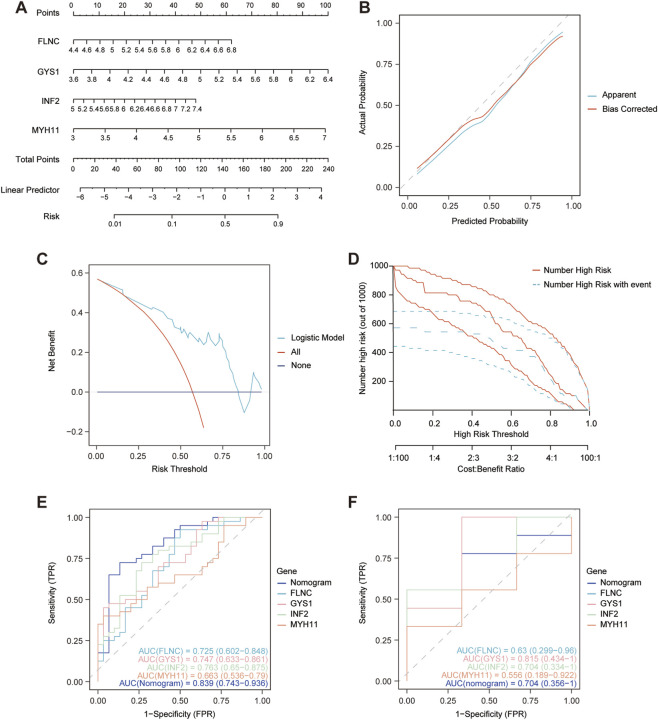
Construction and validation of the nomogram. **(A)** Nomogram incorporating the four core genes. **(B)** Calibration curve for accurate prediction. **(C)** Decision curve analysis (DCA) evaluating net clinical benefit. **(D)** Clinical impact curve demonstrating the predictive performance. **(E, F)** Receiver operating characteristic (ROC) curves assessing discriminatory performance.

Using the four hub genes and a multivariate regression approach, a nomogram model was constructed to predict MCD risk (Figure 5A). Analysis of the calibration curve revealed reasonable agreement between the estimated and actual risks, indicating acceptable calibration performance of the model (Figure 5B). DCA suggested that, within a threshold probability range of 0–0.85, the model may provide net benefits compared with treat-all or treat-none strategies in an exploratory context (Figure 5C). The CIC showed that within the high-risk threshold range (0.4–1), the number of patients predicted to be high-risk showed a trend of correspondence with the actual number of cases, although this finding requires further validation (Figure 5D). The ROC curve analysis indicated that the nomogram model constructed using the four hub genes achieved an AUC of 0.839, suggesting modest exploratory discriminatory ability (Figure 5E). In comparison, the AUC values of the models built using individual genes (0.725, 0.747, 0.763, and 0.663) were all lower, suggesting that the integrated four-gene nomogram exhibited relatively better exploratory performance. Together, these results suggest the exploratory potential of the four-hub-gene signature for further investigation, rather than established clinical utility. Finally, to assess the exploratory prediction capability of the model, an ROC curve was constructed using an external validation dataset (Figure 5F). The model achieved an AUC value of 0.704 in the external validation dataset, indicating limited but non-random predictive capacity that warrants further validation in larger cohorts.

### 3.5 Identification of disease molecular groups

**FIGURE 6:**
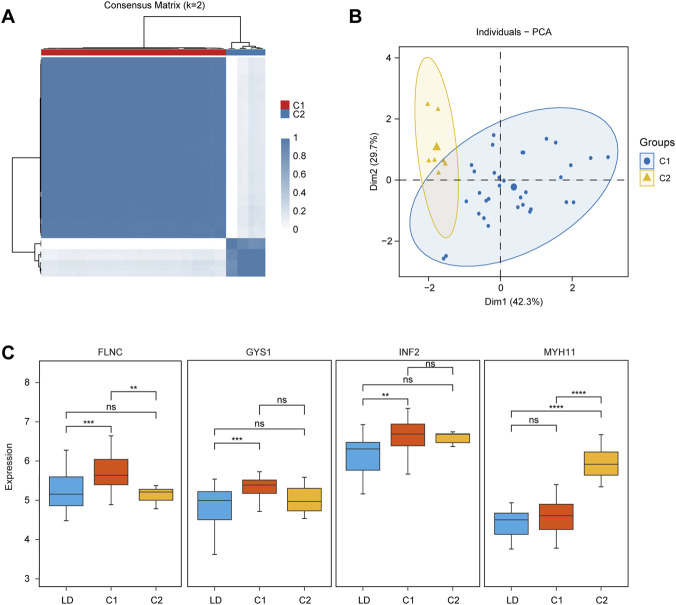
Molecular subtyping of patients with minimal change disease (MCD). **(A)** Consensus clustering matrix for k = 2. **(B)** Principal component analysis (PCA) plot demonstrating the distinct group separation. **(C)** Box plots showing the differential expression of the four core genes across normal controls and MCD groups.

To explore disease heterogeneity, consensus clustering based on the four hub genes stratified patients with MCD into two potential molecular group (C1 and C2). The consensus clustering heatmap revealed minimal sample crossover between the two groups (Figure 6A). Among the 40 patients with MCD, 33 were classified as C1 and 7 as C2. PCA further showed a separation between the two groups (Figure 6B). Gene expression boxplots showed differences in the expression of the four hub genes between C1 and C2, with FLNC, GYS1, and INF2 predominantly upregulated in C1, and MYH11 primarily upregulated in C2, indicating distinct molecular profiles between the two groups (Figure 6C). Because of the higher upregulation of MYH11 in C2, subsequent analyses primarily focused on this gene.

### 3.6 Pathway analysis of molecular group

**FIGURE 7:**
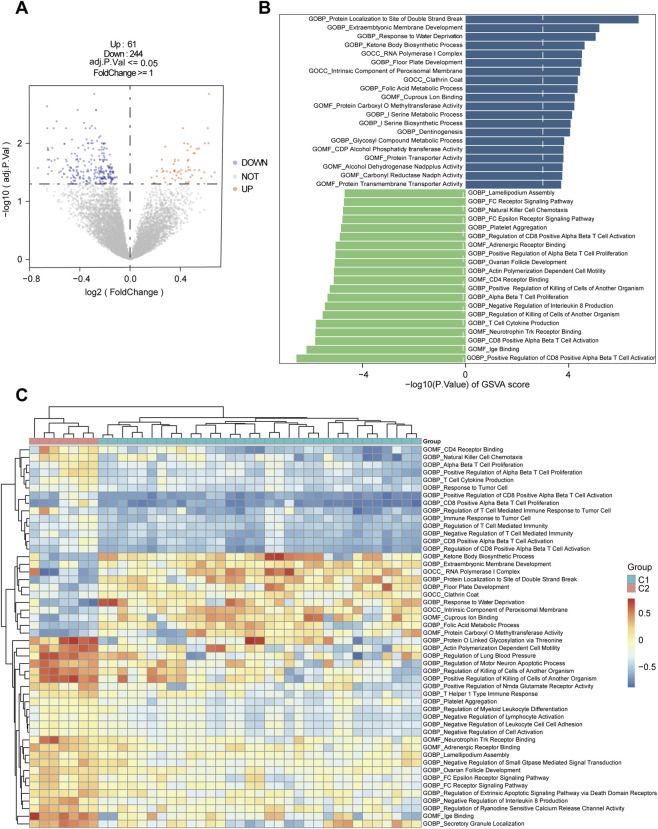
Gene set variation analysis (GSVA) of disease groups. **(A)** Volcano plot of enriched pathways between the C1 and C2 groups. **(B)** Top 20 significantly enriched pathways in each group. **(C)** Heat map of expression patterns of significantly enriched pathways.

We systematically evaluated the pathway–level differences between the two molecular groups of MCD. GSVA enabled the identification of distinct pathways differentially regulated in each group based on their molecular profiles. Volcano plots revealed 305 significantly enriched pathways, with 61 pathways significantly upregulated in the C1 group and 244 pathways significantly upregulated in the C2 group (p < 0.05) (Figure 7A). The enrichment results were visualized using heat maps and bar charts, with the bar charts highlighting the top 20 pathways in both groups (Figure 7B) and the heat maps displaying enrichment scores for the top-ranked pathways in individual samples, providing an intuitive view of the differential regulation (Figure 7C). The enrichment analysis indicated that the C1 showed activation of various metabolic pathways, including small-molecule metabolic pathways, such as ketone body biosynthesis, L-serine metabolism, and folate metabolism. In contrast, the C2 group showed enrichment of immune–related pathways including adaptive immune pathways such as CD8^+^ T cell activation and helper T cell activation, as well as innate immune pathways such as Fc receptor signaling and IL-8 production. Notably, activation of cytoskeleton-related pathways, such as actin polymerization and pseudopod assembly, in this group may reflect immune cell infiltration and glomerular podocyte cytoskeletal abnormalities. Overall, the GSVA results revealed molecular heterogeneity in MCD, consistent with the C1/C2 group classification described above. The association between the C2 group and disulfidptosis aligns with our research focus. As *MYH11* expression was specifically upregulated in this group, it was selected as the primary target for further investigation.

### 3.7 Comprehensive analysis of the immune microenvironment of molecular group

**FIGURE 8:**
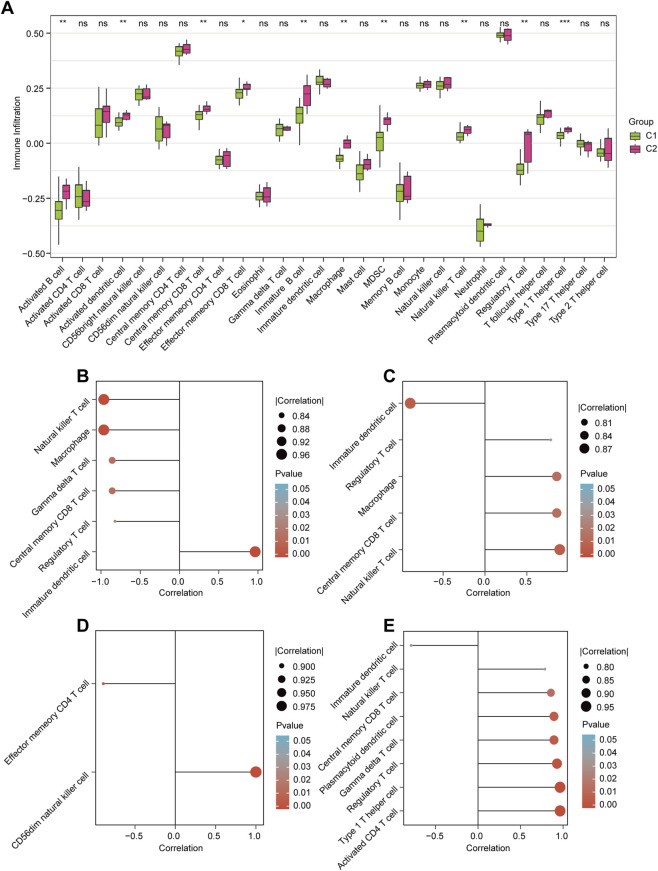
Comprehensive immune microenvironment analysis by group. **(A)** Boxplots of immune cell infiltration between groups. **(B–E)** Lollipop plots showing correlations between *FLNC*/*GYS1*/*INF2*/*MYH11* and immune cells in C2 group.

Immune microenvironment analysis based on ssGSEA revealed notable variations in the infiltration of immune cells across the two molecular groups of MCD. The boxplots showed that the enrichment levels of various immune cells were significantly higher in C2 group than in C1 group, consistent with the GSVA results described above. These findings indicate distinct immune profiles between the two groups (Figure 8A). Because of the prominent immune characteristics of the C2 group, Laplace plots were used to illustrate the correlations between *FLNC*, *GYS1*, *INF2*, and *MYH11* expression and immune cell infiltration in this group (Figures 8B–E). The results revealed correlations between these hub genes and several differentially expressed immune cell types. Notably, *MYH11* exhibited positive correlations with central memory CD8^+^ T cells, natural killer cells, regulatory T cells, and type 1 helper T cells (Figure 8E).

### 3.8 Signaling pathways associated with hub genes

**FIGURE 9:**
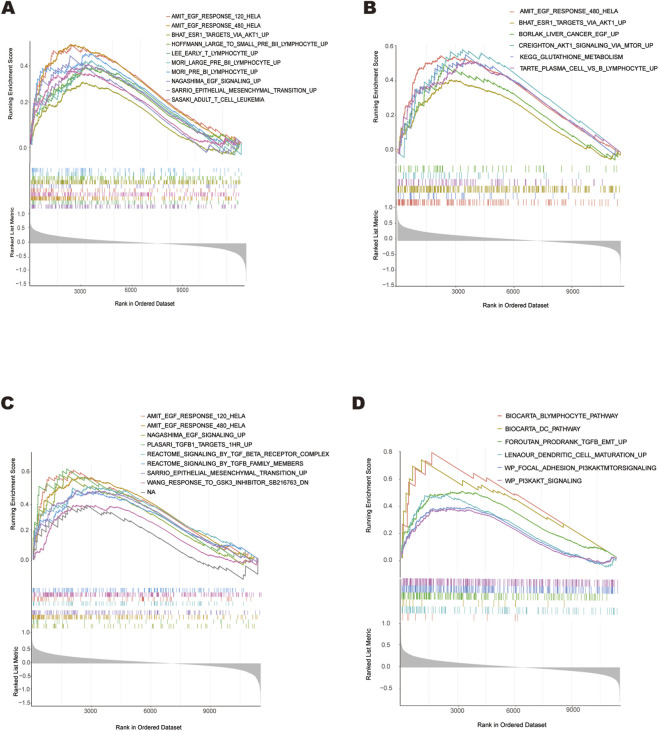
Single-gene-based pathway analysis. **(A–D)** GSEA enrichment plots for pathways associated with *FLNC*, *GYS1*, *INF2*, and *MYH11*, respectively.

To gain deeper insights into the signaling pathways and biological processes linked to the four central genes, we performed sgGSEA. We specifically examined the immune processes and signaling pathways that were significantly correlated with *FLNC*, *GYS1*, *INF2*, and *MYH11*. The sgGSEA results for *FLNC* revealed significant enrichment of multiple T cell- and B cell-related pathways. The enriched immune cell types were consistent with the differentially expressed immune cells identified in the sgGSEA of the disease groups, indicating a potential correlation between *FLNC* expression and pathways related to the immune response. Furthermore, among the upregulated pathways associated with *FLNC*, epidermal growth factor (EGF)- and epithelial–mesenchymal transition (EMT)-related pathways were significantly enriched, revealing a potential role for *FLNC* in the pathogenesis of MCD (Figure 9A). Similarly, *GYS1* showed a significant positive correlation with EGF-related pathways. Notably, glutathione metabolism-related pathways were also significantly upregulated. As aberrant glutathione metabolism is a hallmark of disulfidptosis, these findings reveal a potential link between *GYS1* expression, MCD progression, and disulfidptosis (Figure 9B). The pathways enriched for *INF2* were primarily associated with EGF signaling. Several transforming growth factor (TGF)-related pathways were significantly upregulated, which may be associated with the TGF-induced EMT and its role in the pathogenesis of MCD (Figure 9C). Pathway enrichment for *MYH11* indicated associations with B lymphocyte-, dendritic cell-, and T lymphocyte-related pathways, consistent with the immune features enriched by *FLNC*. In addition, the TGF-induced EMT pathway was significantly enriched among the upregulated *MYH11*-associated pathways, these associations suggest a potential link between *MYH11* expression and immune-related as well as cytoskeleton-related processes in MCD, although further experimental studies are needed to explore these relationships (Figure 9D).

### 3.9 Molecular validation of *MYH11* expression

**FIGURE 10:**
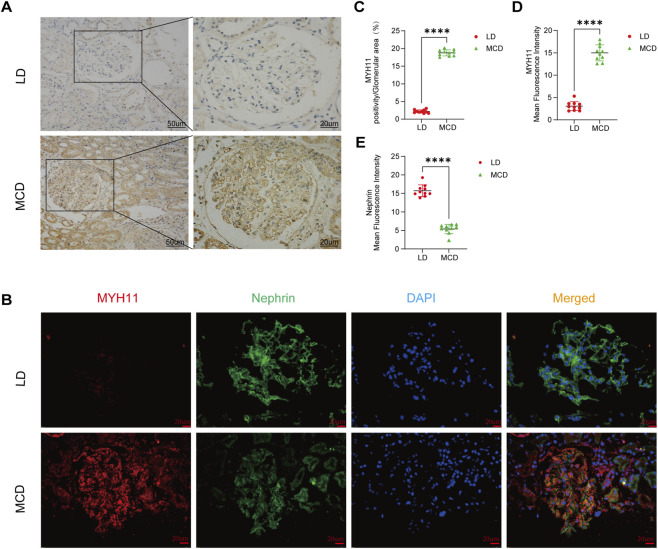
Molecular validation of *MYH11* expression. **(A)** Immunohistochemical staining of *MYH11*. **(B)** Semiquantitative analysis of *MYH11* IHC results. **(C, D)** Semi-quantitative analysis of Nephrin and *MYH11* immunofluorescence. **(E)** Immunofluorescence co-staining for MYH11 and Nephrin.

IHC and IF analyses revealed a significant upregulation of *MYH11* in patients with MCD compared with normal controls, accompanied by a marked reduction in the podocyte-specific marker nephrin (Figures 10A,B). Semi-quantitative and statistical analyses supported these findings, revealing significantly elevated *MYH11* expression (Figures 10C,D) and reduced nephrin levels (Figure 10E) in the MCD group. To evaluate the spatial correlation between MYH11 and nephrin, we conducted co-localization profile analysis. In MCD patients, along a defined linear region traversing the glomerulus, the fluorescence intensity curves of *MYH11* (red) and nephrin (green) showed synchronized fluctuation patterns (Supplementary Figure S3), suggesting spatial proximity within the glomerulus. However, this co-localization does not distinguish whether *MYH11* is expressed in podocytes or in adjacent cell types such as mesangial cells or vascular smooth muscle. The cellular origin of *MYH11* upregulation in MCD remains to be determined in future studies.

## 4 Discussion

MCD represents a significant etiology of nephrotic syndrome across both pediatric and adult populations, characterized by extensive podocyte foot process effacement and the absence of immune complex deposition (Maas et al., 2022; Roman and Nowicki, 2024). Even though its pathogenesis remains unclear, growing evidence implicates podocyte actin cytoskeletal dysregulation (Bărar et al., 2024; Ma et al., 2024). This study explored, for the first time, the potential relationship between disulfidptosis—a novel form of cell-death triggered by abnormal disulfide accumulation leading to actin crosslinking and cytoskeletal collapse—and molecular features of MCD (Zheng et al., 2023; Xiao et al., 2024). Because of the critical dependence of podocyte function on actin stability, we hypothesized that dysregulated expression of disulfidation-related genes was associated with MCD. To investigate this, we performed integrated bioinformatics analysis, including differential expression screening and machine learning, to identify four hub genes and construct an exploratory discriminatory model. Furthermore, unsupervised clustering stratified patients with MCD into two molecular groups (C1 and C2), which exhibited significant differences in gene expression profiles, pathway activity, and immune microenvironment characteristics, highlighting the association between disulfidation-related gene dysregulation and MCD heterogeneity.

Myosin Heavy Chain 11 (*MYH11*), traditionally recognized as a smooth muscle–specific contractile protein, exhibited a notable increase in expression within the glomeruli of patients with MCD in this study. Disruption of actin–myosin organization and the resulting cytoskeletal disarray are known to affect podocyte function (Ma et al., 2024). Our IF data showed that increased *MYH11* signal was spatially associated with reduced nephrin expression, but the exact cell type responsible for *MYH11* upregulation remains unclear. It could involve podocytes, mesangial cells, or vascular smooth muscle cells. Nevertheless, the close spatial association suggests a potential link between *MYH11* and podocyte dysfunction in MCD.

In hepatocellular carcinoma, *MYH11* has been recognized as a candidate transcriptomic marker linked to disulfidptosis (Xu et al., 2023). However, its role in MCD remains unclear. To the best of our knowledge, this study is the first to show significant upregulation of *MYH11* in glomeruli from patients with MCD, suggesting that *MYH11* may be involved in glomerular pathology beyond its traditional smooth muscle function. Because actin cytoskeleton integrity is critical for podocyte function and *MYH11* is known to participate in cytoskeletal organization, we hypothesize that glomerular *MYH11* upregulation could be associated with cytoskeletal remodeling in MCD. Nevertheless, whether this association directly involves podocytes or other glomerular cell types such as mesangial cells or vascular smooth muscle remains to be determined.

Remodeling of the immune microenvironment plays a crucial role in the progression of MCD (Liu and Guan, 2023). GSVA results revealed significant enrichment of immune-related pathways in the C2 group, indicating that alterations in the immune microenvironment may contribute to MCD pathogenesis. Furthermore, subsequent results indicated that the *MYH11*–high C2 group showed enrichment of immune-related features.

ssGSEA revealed that *MYH11* expression was positively correlated with various immune cells, including plasmacytoid dendritic cells and multiple T cell subsets (natural killer T cells, central memory CD8^+^ T cells, gamma delta T cell, regulatory T cells, activated CD4^+^ T cells, and type 1 T helper cells). Notably, except for activated CD4^+^ T cells and gamma delta T cell, the infiltration levels of the remaining T cell subsets were significantly higher in the C2 group than in the C1 group. These findings validated the GSVA results and highlighted the distinct immune profile of the C2 group, indicating that abnormal T cell activation may contribute to MCD pathogenesis. Although the exact mechanism through which *MYH11* shapes the renal immune landscape remains unclear, evidence from oncology has provided valuable insights. In bladder cancer, *MYH11* promotes tumor progression by regulating macrophages, whereas in colorectal cancer, interactions between *MYH11* ^+^ cancer-associated fibroblasts and macrophages are linked to poor prognosis. Moreover, *MYH11* knockdown has been shown to disrupt normal megakaryocyte maturation (Yi et al., 2019; Wang et al., 2024). Meanwhile, a study by Maja Roman and colleagues summarized the role of immune dysregulation in the pathogenesis of MCD(39). Therefore, *MYH11* upregulation in MCD may be associated with remodeling of the intraglomerular immune microenvironment and destabilization of the podocyte cytoskeleton. This potential dual role, impacting both intrinsic podocyte stability and extrinsic immune modulation, positions *MYH11* as a potential factor in MCD pathogenesis. The relationship between *MYH11* upregulation and MCD progression observed in this study aligns closely with these findings, further suggesting an association between *MYH11*, immune microenvironment remodeling, and MCD pathogenesis.

*MYH11* may be associated with the coordinated regulation of multiple signaling pathways that are linked to MCD progression. In vascular diseases, the smooth muscle myosin heavy chain encoded by *MYH11* has been reported to modulate TGF-β signaling (Renard et al., 2013). Consistent with this, the sgGSEA in our study revealed significant upregulation of two TGF-β–related pathways—“WP_TGFBETA_RECEPTOR_SIGNALING_IN_SKELETAL_DYSPLASIAS” and “WP_TGFBETA_RECEPTOR_SIGNALING”—supporting this link. Numerous studies have identified TGF-β as a critical regulator of extracellular matrix production, renal fibrosis, and podocyte injury, and it has been shown to induce EMT in podocytes (Li et al., 2008; Herman-Edelstein et al., 2011; Hills and Squires, 2011). Notably, Kaverina et al. showed that following podocyte depletion in a mouse model, renin–lineage cells underwent mesenchymal–epithelial transition to become podocytes, thereby reducing glomerular injury; during this process, *MYH11* staining as a mesenchymal marker markedly decreased (Kaverina et al., 2017), suggesting that *MYH11* and *TGF-β1* are closely associated with the regulation of the EMT pathway during renal tissue injury. Consistently, our sgGSEA results showed significant upregulation of *MYH11* and TGF-β–relative EMT pathway “FOROUTAN_INTEGRATED_TGFB_EMT_UP” in patients with MCD. Based on these correlational findings, we propose a hypothesis-generating working model (Supplementary Figure S4) in which TGF-β1 signaling and MYH11 expression may be associated, and this association may influence MCD disease progression through the EMT process. However, we emphasize that this model is speculative and that the observed associations do not imply causality. Functional studies, including podocyte TGF-β stimulation, MYH11 knockdown or overexpression, and assessment of podocyte injury markers, are required to test this hypothesis.

GSVA revealed that the C1 group showed enrichment of metabolic reprogramming, including aberrant activation of ketone body metabolism and upregulation of NADP^+^/NADPH–dependent oxidoreductases. This pattern may reflect the metabolic dysregulation and cellular stress responses characteristic of this group and show consistency with the NADPH depletion and disulfide stress observed in disulfidptosis. Differential expression analysis showed significant upregulation of *FLNC*, *GYS1*, and *INF2* in the C1 group, suggesting a potential link with cytoskeletal remodeling in podocytes, a phenomenon that may be associated with the involvement of disulfidptosis in MCD pathogenesis. In contrast, the C2 group showed enrichment of immunological alterations, including significant infiltration of various T cell subsets closely associated with high *MYH11* expression, supporting *MYH11* as a correlative factor in MCD associated with immune microenvironmental abnormalities. Furthermore, the activation of cytoskeleton–related pathways, such as actin polymerization and pseudopod assembly, in the C2 group may be associated with *MYH11*-associated activation of TGF-β pathways in podocytes—an association that involves cytoskeletal remodeling—a process that may be potentially linked to disulfidptosis. These findings highlight distinct regulatory patterns of disulfide-associated pathways among MCD groups and provide a hypothesis-generating framework for understanding disease heterogeneity and the underlying molecular mechanisms of MCD.

Through comprehensive bioinformatic analysis, this study identified four key genes as candidate transcriptomic markers for MCD. These genes may contribute to MCD pathogenesis by regulating the dynamic balance of the podocyte cytoskeleton. Notably, the cytoskeletal remodeling mechanisms associated with these genes showed correlations with the pathological features of disulfidptosis, suggesting that cytoskeletal and metabolic dysregulation may serve as common correlative mechanisms underlying the association between disulfidptosis and MCD progression.

However, this study has certain limitations. First, the sample size used for machine learning and clinical prediction modeling is relatively small. Therefore, the model can only be considered an “exploratory diagnostic tool” and requires further validation in larger-scale cohorts. Second, due to the limited sample size (40 MCD samples with an imbalanced distribution of 33 vs. 7 cases), the stability and sensitivity of the molecular subtyping require further evaluation. We therefore acknowledge that the preliminary results require validation in larger, independent cohorts. Third, the absence of key clinical phenotype data (e.g., proteinuria, steroid sensitivity, treatment response) in the public GEO datasets precludes clinical correlation analysis, making the current findings hypothesis-generating. Future studies with comprehensive clinical data are needed to further validate the findings of this study. Fourth, the experimental validation included only 10 MCD patients and 10 controls, which is a relatively small sample size. Future studies with larger sample sizes are needed to further validate the core findings of this study. Fifth, causal relationships were not established as functional experiments were not performed. Therefore, the proposed working model (Supplementary Figure S4) remains hypothetical and requires validation using cell-based assays such as TGF-β stimulation, *MYH11* knockdown or overexpression, and assessment of podocyte injury markers. Sixth, the cellular source of *MYH11* upregulation in MCD glomeruli remains undefined, as spatial co-localization with nephrin does not confirm podocyte-specific expression. Future studies using single-cell transcriptomics or high-resolution imaging are needed to determine whether *MYH11* originates from podocytes, mesangial cells, or other glomerular cell types.

## 5 Conclusion

Overall, these results help to improve the comprehension of the molecular features underlying MCD, particularly *MYH11*, for future investigation. Using integrated bioinformatics and multi-method screening, this study identified four disulfidptosis-related genes as candidate transcriptomic markers for MCD. Patients were classified into two molecular groups. These two preliminary molecular groups exhibit distinct pathway enrichment patterns: C1 is characterized by enrichment of metabolic pathways, and C2 by enrichment of immune-related pathways and cytoskeletal remodeling. These findings suggest an association between disulfidptosis-related gene signatures and MCD pathogenesis and provide exploratory insights that may inform future hypothesis-driven studies. The proposed working model involving *MYH11* should be considered speculative and requires experimental validation.

## Data Availability

RNA-Seq data was downloaded from the GEO database under Project Accession No. GSE108109, GSE200828, and GSE104948. Data supporting the findings of this study are available from the corresponding authors upon request. All analysis code is available at: https://github.com/LLL-art-svg/code.

## Supplementary material

SUPPLEMENTARY FIGURE S1 Assessment of batch-effect correction. **(A)** Boxplot of gene expression values before batch correction. **(B)** Boxplot after Limma-based batch correction. **(C)** PCA plots after batch correction.

SUPPLEMENTARY FIGURE S2 Functional enrichment analysis of the overlapping genes between differentially expressed genes (DEGs) and WGCNA moudle genes in MCD. **(A)** Veen diagram showing intersections among DEGs and WGCNA module genes. **(B)** Bubble plot of the GO enrichment analysis for the overlapping genes.

SUPPLEMENTARY FIGURE S3 Co-localization profile analysis of MYH11 and nephrin. A) Fluorescence intensity profiles of MYH11 (red) and nephrin (green) along a linear region acrossan MCD glomerulus.

SUPPLEMENTARY FIGURE S4 A hypothesis-generating working model illustrating potential associations between TGF-β signaling and *MYH11* expression in the glomerulus. Dashed arrows indicate speculative associations that require experimental validation. The schematic illustrates a hypothesized molecular cascade in which TGF-β signaling activation is associated with increased *MYH11* expression, potentially contributing to nephrin downregulation, cytoskeletal remodeling, and epithelial-mesenchymal transition (EMT)-like changes. The cellular source of *MYH11* in this model is not definitively determined by the current data and may involve podocytes, mesangial cells, or other glomerular cell types.
