# Supplementary figures and images for: An exploratory analysis of disulfidptosis-related gene signatures in minimal change disease identifies metabolic and immune associations
Source: Front Cell Dev Biol. 2026 Jun 19;14:1790068. doi: 10.3389/fcell.2026.1790068 (PMC13328497; doi:10.3389/fcell.2026.1790068)

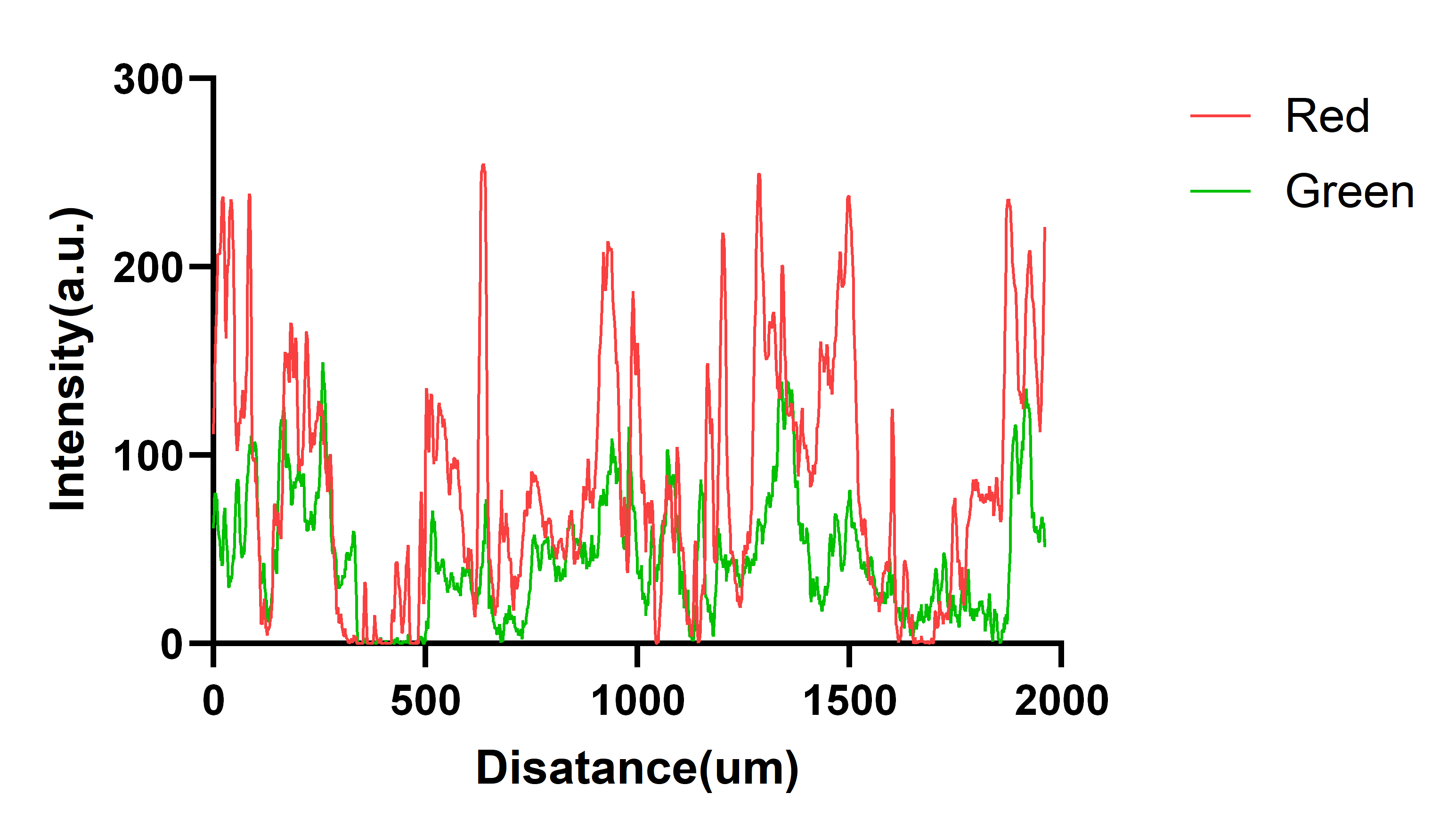

Supplement: Supplementary file 1 [file Image3.tif]

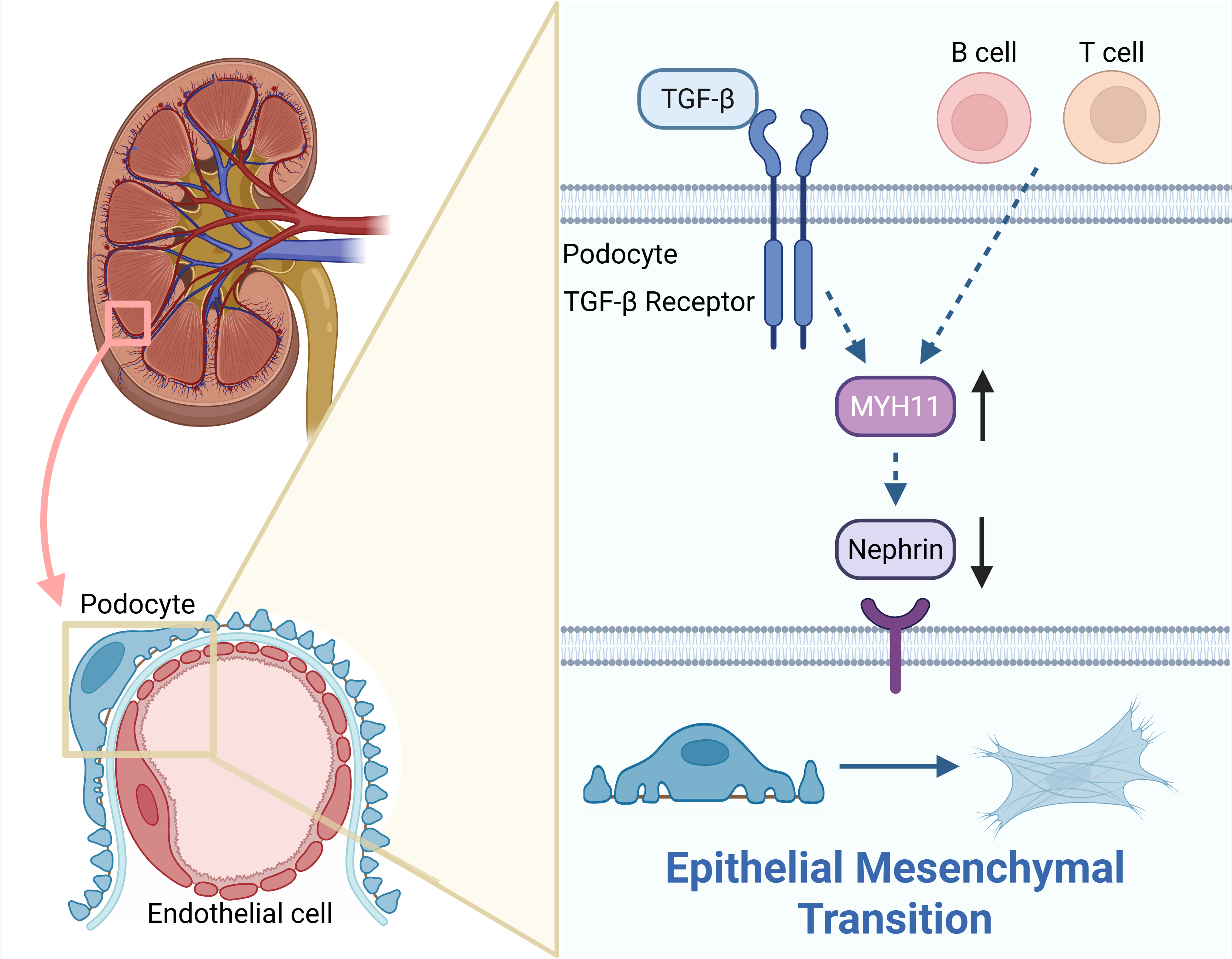

Supplement: Supplementary file 2 [file Image4.jpeg]

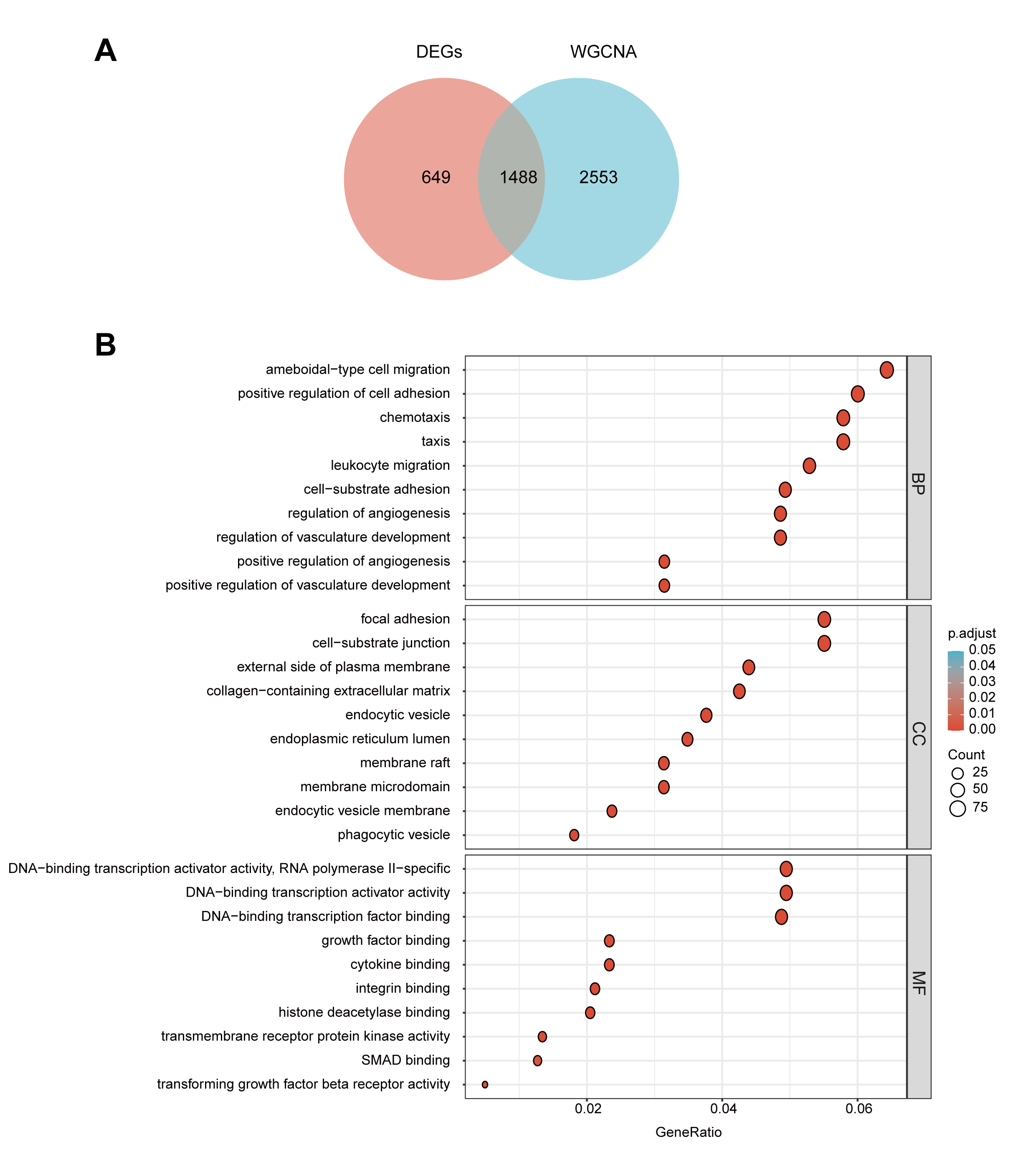

Supplement: Supplementary file 3 [file Image2.tif]

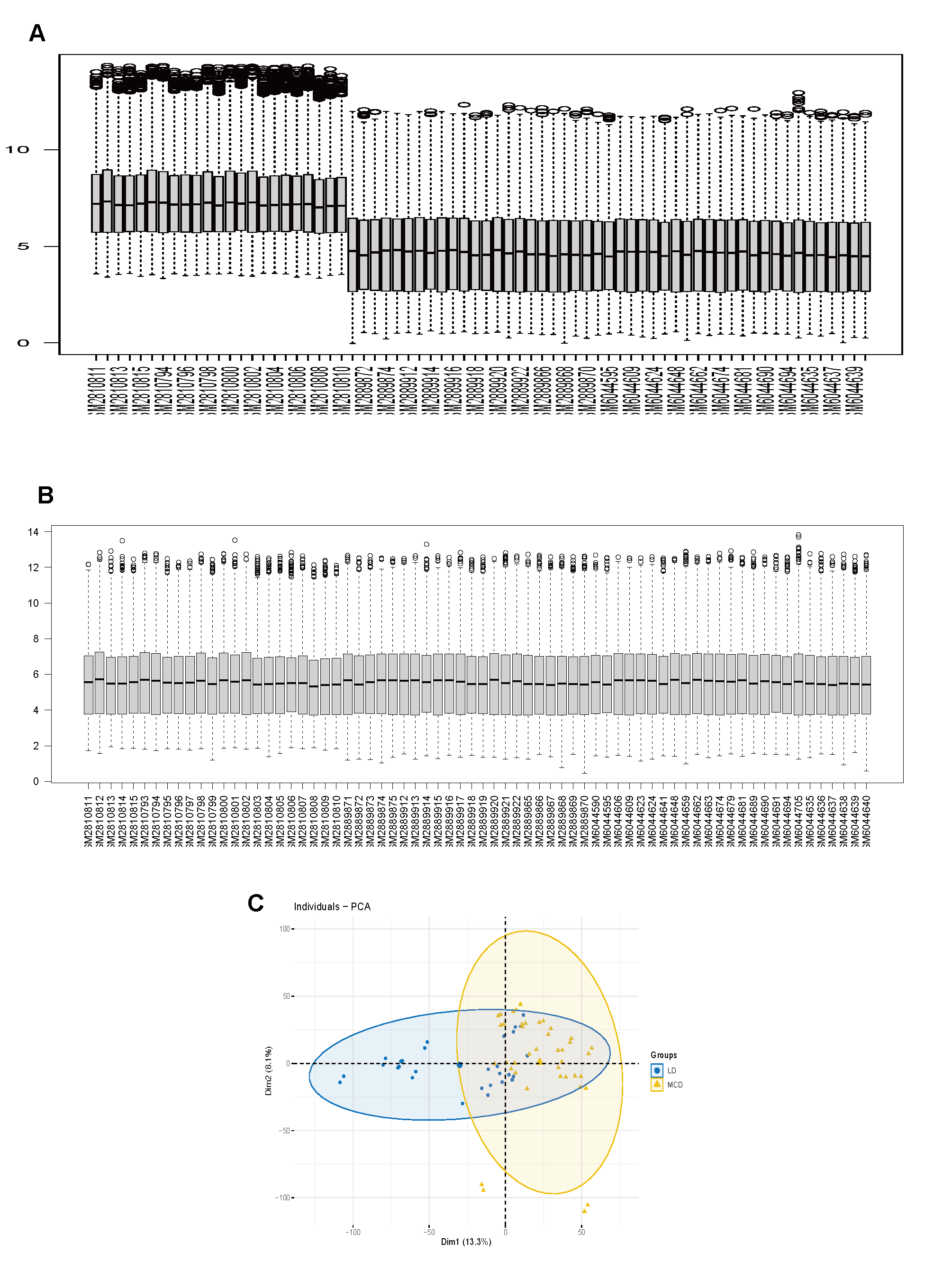

Supplement: Supplementary file 4 [file Image1.tif]
